# Extracts from the Records of the Chicago Academy of Medical Sciences

**Published:** 1859-08

**Authors:** S. C. Blake

**Affiliations:** Secretary


### PROCEEDINGS OF MEDICAL SOCIETIES.

### EXTRACTS FROM THE RECORDS OF THE CHICAGO ACADEMY OF MEDICAL SCIENCES.

S. C. BLAKE, M. D., *Secretary.*

May 6th, 1859.

Dr. L. P. Cheney was duly elected a fellow of the Academy.

Dr. Orrin Smith read an interesting paper on the pathology and treatment of croup. Dr. S. believes croup to be an ordinary inflammation, deriving its alarming and fatal character from the locality of the lesion; hence, the treatment should be antiphlogistic. He depends with great confidence upon antimony, mercury and veratrum viride; and his success, during a long series of years of extensive and varied practice, has been such that he no longer looks upon this terror of physician and parent as necessarily alarming, providing the physician is called in time to procure the specific effects of the remedies before a fatal termination.

Prof. J. H. Rauch presented the Academy with a valuable donation of specimens of the indigenous medicinal plants of the North-west.

Dr. E. L. Holmes read a very interesting paper on a new method of aiding vision in cases of opacity of the cornea.

Drs. Ross, Bloodgood and Cheney were appointed a committee to report to the Academy a new fee bill.

June 6th, 1859.

Dr. V. L. Hurlbut reported an interesting case of puerperal convulsions, which resulted in recovery.

Patient aged 21. When two months advanced in her first pregnancy, she was violently threatened with premature labor. At this time had some hemorrhage, but was relieved by the following prescription:

℞ Viburen,
Scutellarin,
Caudophyllin, aa. gr. viij.
Gelsemin, gr. iv., ft. pil. No. xvj.

Take two pills every one or two hours till relieved.

Dr. H. would call the attention of the profession to the active principles of vegetable medicines, especially the above articles, in whose power to relieve pain and allay nervous irritability, a long experience with them has given him great confidence.

When patient was seven months advanced in pregnancy, March 18th, Dr. H. was called, and found her suffering from severe uterine pains, which were quite regular; os uteri dilated, so as to readily admit the end of the index finger. Ordered the above prescription, and in one hour and a half patient was entirely relieved from pain.

*March 20th.* Found patient suffering from slight pain in top of head; had rested well the night previous; pulse 85; tongue with brown coat; bowels constipated, having had no dejection for one week. Ordered an enema to be given immediately.

In about an hour and a half from the last visit, in the absence of Dr. H. his father was called and found patient just recovering from a convulsion, with retching and vomiting; pulse small and quick; dark coating on tongue; head hot; feet inclined to be cold. Ordered calomel gr. i., to alloy vomiting, and in half an hour gave the following:

℞ Calomel, gr. xv.
Sodæ sub. carb., gr. viij.

with drafts to feet and cold applications to head.

Dr. H. returned in half an hour and found patient had vomited medicine. Stomach irritable and unable to retain anything. Ordered an enema, which was followed by a large, dark colored and very offensive dejection.

Convulsions continued for thirteen hours to the number of twenty, coma and stertorous breathing filling up the intervals.

After long continued and careful manipulation by Dr. H., senior, the os uteri was sufficiently dilated to deliver.

Prof. Wm. H. Byford was called in consultation. At this time pains had entirely ceased; pulse 216.

Prof. B. proceeded to extract with the crotchet, after having made an unsuccessful attempt to deliver with the forceps.

Patient slowly recovered, having no convulsions after delivery.

Dr. J. N. Graham read a very interesting paper on medicinal inhalation in the treatment of chronic diseases of the throat and lungs. Dr. G. reported several cases of bronchitis and phthisis in its different stages, in the treatment of which he had been able to afford his patients great benefit. In many cases of the former disease he had succeeded in effecting a cure, and in the latter of alleviating much suffering, and of prolonging the life of his patients. Dr. G. uses principally iodine, iodide of potassium, cyanuret of potassium and hydrocyanic acid. He uses with the remedies tincture conium and chloroform, to allay irritation.

Dr. Ingals thought inhalations very good in cases of phthisis, but he believed the inhalation of pure air, accompanied with moderate exercise, if not the only remedy from which to expect benefit, very much the most important. Dr. I. would recommend to a patient with phthisis to purchase a horse and saddle and make a trip to Texas for a drove of cattle.

July 4th, 1859.

Dr. E. Ingals read a very interesting and able report of the reduction of a luxation of the head of the humerus of six weeks' standing. The operation resulted in an entire success; the joint and muscles in its vicinity being restored to their normal condition in two months, with the exception of slight atrophy of the deltoid.

Dr. Ingals thought all such cases as the above should be reported, that the profession may have some data from which to decide at what length of time from the dislocation such reduction may with safety be undertaken.

